# Acceptability of psychosocial interventions for dementia caregivers: a systematic review

**DOI:** 10.1186/s12888-018-1976-4

**Published:** 2019-01-14

**Authors:** Dan Qiu, Mi Hu, Yu Yu, Bingwei Tang, Shuiyuan Xiao

**Affiliations:** 1 Department of Social Medicine and Health Management, School of Public Health, Central South University, 110 Xiangya Road, Changsha, 410078 Hunan China; 2 Hospital Evaluation Office, Xiangya Hospital, Central South University, Xiangya Road 87, Changsha, 410008 Hunan China

**Keywords:** Acceptability, Caregivers, Dementia, Psychosocial interventions

## Abstract

**Background:**

Most of patients with dementia are cared for by family members. Caring for people with dementia is challenging; approximately 30–55% of caregivers suffered from anxiety or depressive symptoms. A range of studies have shown that psychosocial interventions are effective and can improve caregivers’ quality of life, reduce their care burden, and ease their anxiety or depressive symptoms. However, information on the acceptability of these interventions, despite being crucial, is under-reported.

**Methods:**

Systematic searches of databases were conducted for literature published on EMBASE, PubMed, The Cochrane Library, Web of Science, and PsycARTICLES until August 2017 and the searches were updated on June 2018. The selection criteria included primary studies with data about the acceptability of psychosocial interventions for informal caregivers and publications written in English. Two authors independently selected studies, extracted study characteristics and data, assessed the methodological quality of the included studies by using the Effective Public Health Practice Project (EPHPP) Quality Assessment Tool and Critical Appraisal Skills Programme (CASP) Qualitative Research Checklist, and conducted a narrative synthesis of quantitative and qualitative data.

**Results:**

A total of 10,610 abstracts were identified through systematic searches. Based on screening titles and abstracts, 207 papers were identified that met the criteria for full paper review, with 42 papers from 13 different countries meeting the inclusion criteria. We found high- and moderate-quality evidence showing psychosocial interventions were acceptable, with important benefits for caregivers. Facilitators of acceptability included caregivers’ need for intervention, appropriate content and organization of the intervention, and knowledge and professionalism of the staff. Barriers to acceptability included participants’ poor health status and low education levels, caregiving burden, change of intervention implementers, and poor system performance of interventions.

**Conclusion:**

There is preliminary evidence to support the acceptability of psychosocial interventions for dementia caregivers. However, the available supporting evidence is limited, and there is currently no adequate information from these studies indicating that the acceptability has received enough attention from researchers. More well-designed studies assessing psychosocial interventions are needed to give specific statements about acceptability, and the measure of acceptability with psychosocial interventions should be more comprehensive.

**Electronic supplementary material:**

The online version of this article (10.1186/s12888-018-1976-4) contains supplementary material, which is available to authorized users.

## Background

Caring for people with dementia is challenging, as most patients may lose the ability to understand and communicate effectively, and general care hardly improves their condition [1]. Compared with caregivers in other conditions, dementia caregivers provide more assistance, give up their vacations or hobbies more often [2–4], and have a higher risk of becoming physically and mentally ill [4, 5]. Marcia et al.’s studies have shown that 55% of dementia caregivers had to give up pleasurable personal activities [4], and about 30–55% of caregivers suffered from anxiety or depressive symptoms [6–8], which may have resulted in changes in their behavior and reduced quality of care.

Derived from wide-ranging theories and concepts, psychosocial interventions mean physical, cognitive or social activities that aim at addressing a specific health or social care problem [9, 10]. There are a range of studies showing that psychosocial interventions are moderately effective for dementia caregivers, which can improve caregivers’ quality of life, reduce their care burden, and ease their anxiety or depressive symptoms [9, 11–15]. However, those psychosocial interventions often encounter many difficulties. For example, although understanding the needs and situations of caregivers is valuable and crucial for developing effective intervention plans, few programs systematically assess caregiver needs [16]. Coupled with a number of other constraints, such as transportation barrier, time constraints, and stigma [17], many caregivers refused to participate in the intervention. Moreover, many trials neither had a practice manual nor specific description about the process of interventions, making them difficult to be reliably replicated in practice [18]. Additionally, training for intervention implementers is often difficult to complete or not widely available under the constraints of many factors [18]. Moreover, due to differences in culture, economic development and social resources, few psychosocial interventions can be widely accepted and implemented among caregivers in different regions [19].

As a construct in public health practice, acceptability means the reaction of intended recipients and refers to judgments by participants and others on whether intervention procedures are appropriate, fair, and reasonable for the problem or participants [20, 21]. When translating the results from trials to practice, a key component in the uptake and success of an intervention is its acceptability to targeted individuals, which may have direct implications for its dissemination and utilization [22, 23]. Many studies in this field do not measure acceptability directly but report the following indicators as acceptability of psychosocial interventions: take-up rates, drop-out rates, and caregivers’ evaluation of the intervention (e.g., satisfaction, preference) [22, 24–26]. To develop effective interventions for caregivers of people with dementia, it is important to understand whether the intervention is acceptable in practice and what elements and which methods are acceptable. The current review aims to systematically assess evidence for the acceptability of psychosocial interventions for informal caregivers of people with dementia and to summarize the factors related to the acceptability of psychosocial interventions. The findings in this review may inform the development and implementation of future research and enhance the application value of psychosocial interventions for dementia caregivers.

## Methods

### Search strategy

We searched the Web of Science, EMBASE, the Cochrane Library, PubMed, and PsycARTICLES with no restrictions on date or language of publication up until 18 August 2017 and conducted an update search on 02 June 2018. Search terms were gathered into four facets: dementia (including dementia, Alzheimer Disease, Vascular Dementia, Huntington Disease, etc.), informal caregiver (including carer*, caregiver*, family relations, etc.), psychosocial intervention (including psychosocial/psychological/non-pharmacological intervention*, family intervention, psycho-education*, cognitive, psychotherapy, social/emotional/information support, exercise, etc.), and acceptability (including acceptability, adherence, compliance, satisfaction, utilization, etc.). See Additional file 1 for a full search strategy.

### Inclusion/exclusion criteria

Inclusion criteria

We included studies that fulfilled all the following criteria:

● Participants: family, informal, unpaid caregivers of patients with dementia or dyads (dementia patients and their informal caregivers).

● Intervention: any psychosocial interventions delivered to informal caregivers of people with dementia, aimed at improving their function or quality of life or providing support.

● Outcome: participation rate, completion rate, or caregivers’ evaluation of the intervention (satisfaction, preference, etc.).

● Study design: quantitative or qualitative studies.

Exclusion criteria

We excluded studies if:

● The study was not in English.

● The study included both informal and formal caregivers.

● The report was a review, meta-analysis, conference abstract, pilot study, letters, or protocol.

### Quality assessment

The methodological quality of the included studies was independently assessed by two reviewers (DQ and MH) using the Effective Public Health Practice Project (EPHPP) Quality Assessment for quantitative studies [27–29], which enables an assessment of selection bias, study design, use of blinding, the level of confounding, data collection methods and data analysis. For qualitative studies, we used the Critical Appraisal Skills Programme (CASP) checklist [30], which includes 10 aspects, such as appropriateness of the methodology, research design, recruitment strategy, data collection, ethical issues, and influence of the relationship between researcher and participants. See Additional file 2 for details on the quality assessment.

### Data extraction and synthesis

Data were extracted on country of origin, year of publication, study design, sample size, workforce, setting, and measures of acceptability independently by two reviewers (DQ and MH). The qualitative and quantitative data of included studies were combined in a synthesis [31–33]. The results were grouped, where possible, by two analytical themes: (i) the acceptability of psychosocial interventions, included take-up rates, drop-out rates, and caregivers’ evaluation of the intervention, (ii) factors related to the acceptability of psychosocial interventions, included facilitators of acceptability and barriers to acceptability. In the initial analysis, only high-quality and medium-quality articles were included; when the evidence was not sufficient, the low-quality articles were analyzed. Consensus was reached on discrepancies in data extraction through discussion.

## Results

### Study selection

**Fig. 1:**
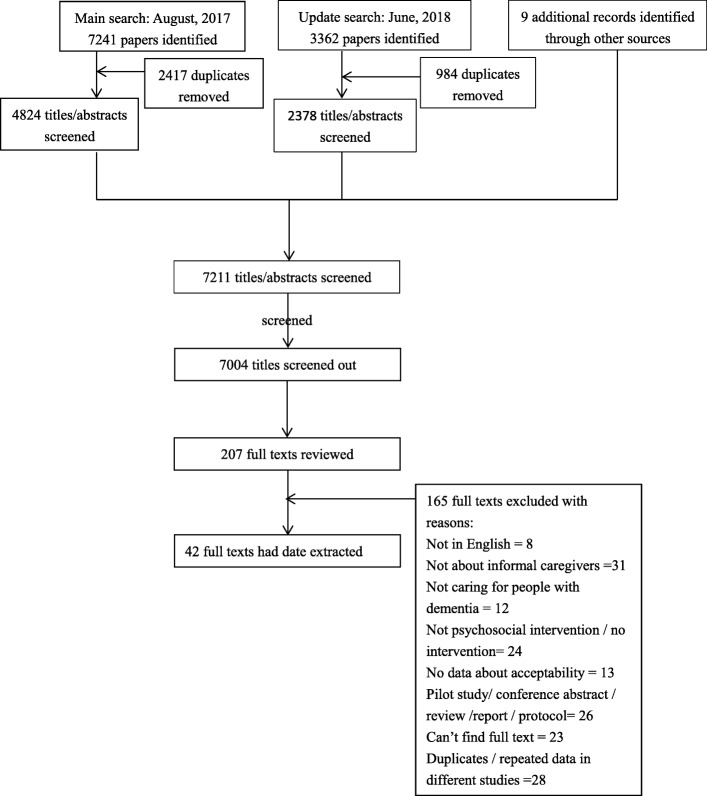
Flowchart of the search strategy

A total of 10,610 references were identified. After excluding duplicates, 7211 articles were screened. By screening titles and abstracts, 207 papers were identified that met the criteria for full paper review. Among them, 43 were excluded for not about dementia or informal caregivers, 13 were excluded for not reporting data on acceptability, 24 were excluded for not psychosocial interventions, 8 were excluded for not in English, 28 were excluded for repeated data, 23 were excluded for no full text available and 26 were excluded because they are not original studies (reviews, conference abstract, protocol, etc. Finally, a total of 42 eligible articles were included in this systematic review (Fig. 1).

### Characteristics of studies

The included studies were from 13 countries in North America, Europe, Asia and Oceania. The studies presented a wide variety of designs, including 29 randomized controlled trials, 8 pre-test /post-test or quasi-experimental designs, 3 qualitative studies and 2 mixed-method designs. Ten of the included studies were on cognitive activities, 2 on physical activities, 6 on social activities and 24 on other psychosocial interventions (such as case management, combined physical and cognitive activities, combined social and psychological activities, et al.). Details about interventions in the studies are presented in Additional file 3. Workforce and settings of interventions are described in Table 1.

**Table 1:** Summary characteristics of included studies

Variable	Number of studies	%
Study design		
Randomized controlled trial	29	69%
Pre-test/post-test trial or quasi-experiment	8	19%
Mixed method	2	5%
Qualitative	3	7%
Intervention target		
Caregiver	23	55%
Dyads (Patient and caregiver)	19	45%
Setting		
Home-based	22	52%
Hospital/outpatient clinics	6	14%
Community health center	2	5%
Social settings (shops, church, etc.)	1	2%
Telephone/internet-based	7	17%
Not reported	4	10%
Implementation workforce		
Specialist	37	88%
Lay worker	2	5%
Not reported	3	7%
Intervention type		
Cognitive	10	24%
Physical	2	5%
Social	6	14%
Other psychosocial intervention (multi-component)	24	57%

### Quality assessment

We completed 44 quality assessments on the 42 included studies; two studies [34, 35] with mixed-methods design each had 2 quality assessments done. Details of the methodological quality assessments of all 42 studies are in Additional file 2. From the 42 papers, 21 (50%) studies were rated as strong [17, 36–55], 15 (36%) were rated as moderate [24–26, 35, 56–66], and 6 (14%) were rated as low [34, 67–71]. Most of the included studies used data collection tools with high reliability and validity, and differences in the quality assessment arose mainly from sample representativeness, drop-out rates, integrity of intervention, and appropriateness of the analysis.

### Results of data synthesis

Three themes for acceptability of the interventions emerged: recruitment and participation rate, completion rate, and participants’ satisfaction with the intervention [22, 72]. Two themes on factors related to the acceptability of interventions emerged: facilitators of acceptability and barriers to acceptability. Of the 40 included studies that had data about the acceptability of the interventions, 13 studies showed either quantitative or qualitative data about factors related to the acceptability of interventions. Details about the acceptability of the interventions in the studies are described in Table 2.

**Table 2:** Acceptability of the interventions

Outcome	Study	Participants	Type of intervention	Rate/score
Participation rate	Mahoney [36], 2001	Caregivers	Social intervention, include Monitoring and counseling, support, and Activity/caregiver respite conversation.	84.7%
	Hebert [57], 1994	Caregivers	Social intervention, include dementia education, behaviour or emotional Problem-solving, etc.	52.9%
	McCurry [61], 2015	Caregiver	Cognitive intervention, include Problem-solving skills, Effective communication skills, negative thinking management, Coping strategies, education.	95.6%
	Wilz [48], 2016	Caregiver	Cognitive Behavioral Therapy	88.4%
	Rotrou [50], 2011	Dyads	Cognitive intervention, Include education, problem-solving techniques, emotion-centred coping strategies, behavior management, communication skills, crisis management, practical advice	95%
	Pitkala [45], 2011	Dyads	Physical intervention, tailored home-based exercise	94%
	Castro [59], 2002	Caregivers	Physical intervention, exercise program	41.2%
	Mittelman [55], 2007	Caregivers	Individual and family counseling, Provide counseling depended on the needs of each spouse caregiver and family	99%
	Barbabella [35], 2018	Caregivers	Web-based psychosocial intervention, Psychological and social support, Coping and reconciliation strategies, education, contact service.	95.9%
	Zarit [43], 2013	Caregivers	Social and psychological intervention, Advanced Caregiver Education and Support Program	90%
	Chee [46], 2007	Caregivers	Skill-Building Intervention, include education, problem solving, communication, environmental and task simplification techniques, etc.	88%
	Belle [52], 2006	Caregivers	Multi-component intervention, include problem solving, skills training, stress management techniques, and telephone support groups, et al.	67%
	Tremont [17], 2015	Caregivers	Psychosocial intervention, include dementia education, emotional support etc.	66.8%
	Livingston [47], 2014	Caregivers	psychological intervention, START manual-based, individual coping intervention	55%
	Whitlatch [25], 2006	Dyads	multi-component EDDI dyadic program, include emotional support, communication skills, Dementia education	91%
	Eloniemi-Sulkava [54], 2009	Dyads	Multi-comonent support program, include goal-oriented peer support group meetings, dementia education,	86%
	Orsulic-Jeras [60], 2016	Dyads	Social and psychological intervention, Dyadic counseling-based, include support activities, and education.	82%
	Burgio [49], 2003	Dyads	Psychosocial intervention, include skills training program	69%
	Vickrey [67], 2006	Dyads	Care management intervention, include problem-solving skills, and provide supports based on their need.	43%
	Woods [65], 2016	Dyads	RYCT program intervention, multi-component intervention.	36%
	Joling [67], 2013	Dyads	Multi-component family meetings intervention, include psycho-education, problem solving techniques, emotional and instrumental support	31.9%
Completion rate	Winter [63], 2006	Caregivers	Social intervention, providing emotional support, coping strategies.	91.3%
	Mahoney [36], 2001	Caregivers	Social intervention, include Monitoring and counseling, support, and Activity/caregiver respite conversation.	97%
	Xiao [37], 2015	Caregivers	Social intervention, Based on caregivers’ need, provide face-to-face coaching and support	84.7%
	Hebert [57], 1994	Caregivers	Social intervention, include dementia education, behaviour or emotional Problem-solving, ect.	80%
	Mohide [65], 1990	Caregivers	Social intervention, help the caregivers enhance caregiving competence and achieve a sense of control in their roles as caregivers	70%
	Eloniemi-Sulkava [54], 2009	Dyads	Multi-component support program, include goal-oriented peer support group meetings, dementia education,	100%
	Martin-Carrasco [40], 2009	Caregivers	Cognitive intervention, include behavioural problems handing strategies, tension and stress control, dementia education.	90.4%
	Wilz [48], 2016	Caregivers	Cognitive intervention, Cognitive Behavioral Therapy	79.5%
	Callan [70], 2015	Caregivers	Cognitive intervention, provide information about dementia, social support, coping skills training, affective self-management, and healthy sleep practices.	74%
	McCurry [61], 2015	Caregivers	Cognitive intervention, include Problem-solving skills, Effective communication skills, negative thinking management, Coping strategies, education.	64%
	Pot [38], 2015	Caregivers	Cognitive intervention, include Psycho-education, Problem-solving, Behavioral activation, Time-management, Cognitive restructuring.	45.6%
	Liddle [69], 2012	Dyads	Cognitive intervention, include memory and communication training support.	76%
	Rotrou [50], 2011	Dyads	Cognitive intervention, Include education, problem-solving techniques, emotion-centred coping strategies, behavior management, communication skills, crisis management, practical advice	69.7%
	Leung [62], 2017	Dyads	Cognitive intervention, individual cognitive stimulation therapy.	61%
	Castro [59], 2002	Caregivers	Physical intervention, exercise program	74%
	Pitkala [45], 2011	Dyads	Physical intervention, tailored home-based exercise	92%
	Dahlrup, B [68], 2014	Caregivers	Social and psychological intervention, include dementia education, support group.	85%
	Tremont [17], 2015	Caregivers	Psychosocial intervention, include dementia education, emotional support etc.	85%
	Barbabella [35], 2018	Caregivers	Web-based psychosocial intervention, Psychological and social support, Coping and reconciliation strategies, education, contact service.	79.7%
	Coon [58], 2003	Caregivers	Psycho-educational skill training interventions, include Anger and depression management class	77%
	Livingston [47], 2014	Caregivers	Psychological intervention, START manual-based, individual coping intervention	75%
	Belle [52], 2006	Caregivers	Multi-component intervention, include problem solving, skills training, stress management techniques, and telephone support groups, et al.	75%
	Chee [46], 2007	Caregivers	Skill-Building Intervention, include education, problem solving, communication, environmental and task simplification techniques, etc.	74%
	Mittelman [55], 2007	Dyads	Individual and family counseling, Provide counseling depended on the needs of each spouse caregiver and family	97.5%
	Laakkonen [44], 2013	Dyads	Self-management group intervention	93%
	Kwok [53], 2012	Dyads	Case management, include home visits, telephone supports, et al.	90%
	Burgio [49], 2003	Dyads	Psychosocial intervention, include skills training program	84.3%
	Vickrey [66], 2006	Dyads	Care management intervention, include problem-solving skills, and provide supports based on their need.	82%
	Jansen [56], 2011	Dyads	Case management, include 2 home visits, personal care plan, family-meeting, etc.	81.8%
	Søgaard [51], 2014	Dyads	Multi-component intervention, include disease education, communication skills, et al.	80%
	Prick [24], 2014	Dyads	Multi-component psychosocial intervention, exercise and support	77.2%
	Joling [67], 2013	Dyads	Multi-component family meetings intervention, include psycho-education, problem solving techniques, emotional and instrumental support	77%
	Roberts [34], 2009	Dyads	Social and psychological intervention, include individual and family consultations, support group, weekly four-session education	74%
	Whitlatch [25], 2006	Dyads	Multi-component EDDI dyadic program, include emotional support, communication skills, Dementia education	65%
	Orsulic-Jeras [60], 2016	Dyads	Social and psychological intervention, Dyadic counseling-based, include support activities, and education.	65%
	Woods [64], 2016	Dyads	Multi-component intervention, RYCT program.	57%
Satisfaction with the interventions	Pot [38], 2015	Caregivers	Cognitive intervention, include Psycho-education, Problem-solving, Behavioral activation, Time-management, Cognitive restructuring.	The average satisfaction of caregivers was 4.16(5 = highest satisfaction
	Beauchamp [39], 2005	Caregivers	Cognitive intervention, include knowledge, cognitive, and behavioral skills training.	The average satisfaction of caregivers was 5.1(7 = highest satisfaction
	McCurry [61], 2015	Caregivers	Cognitive intervention, include Problem-solving skills, Effective communication skills, negative thinking management, Coping strategies, education.	92% were satisfied with the intervention
	Martin-Carrasco [40], 2009	Caregivers	Cognitive intervention, include behavioural problems handing strategies, tension and stress control, dementia education.	The caregivers that program was ‘useful’ or ‘very useful’ once the PIP had finished (97.7%), and 6 months later (93.2%)
	Gaugler [71], 2015	Caregivers	Cognitive intervention, Psycho-education program	More than 90% of family caregivers strongly agreed or agreed that CARES for Families was clear, easy to understand, and improved confidence in dementia care
	Wilz [48], 2016	Caregivers	Cognitive intervention, Cognitive Behavioral Therapy	Caregivers from the CBT group evaluated the telephone setting as very good (71.9%) and 27% as good.
	Xiao [37], 2015	Caregivers	Social intervention, Based on caregivers’ need, provide face-to-face coaching and support	95.9% were satisfied with the intervention
	Czaja [42], 2013	Caregivers	Multi-component psychosocial intervention, include problem-solving strategies, stress management, communication strategies, healthy behavior strategies	73% indicated that they benefitted a great deal from participating in the project
	Zarit [43], 2013	Caregivers	Social and psychological intervention, Advanced Caregiver Education and Support Program	94% caregivers were satisfied with the intervention
	Tremont [17], 2015	Caregivers	Psychosocial intervention, include dementia education, emotional support etc.	The average satisfaction of caregivers was 3.84(4 = highest satisfaction)
	Liddle [69], 2012	Dyads	Cognitive intervention, include memory and communication training support.	Caregivers said the training was perceived to be very useful (85%, *n*=11) or fairly useful (15%, *n*=2)
	Puranen [41], 2014	Dyads	Nutritional intervention, include tailored nutritional advice, home visit etc.	93% estimated that the intervention were useful for them
	Jansen [56], 2011	Dyads	Case management, include 2 home visits, personal care plan, family-meeting, etc.	Overall, caregivers were satisfied with the quality of the intervention
	Whitlatch [25], 2006	Dyads	Multi-component EDDI dyadic program, include emotional support, communication skills, Dementia education	Caregiver ratings of treatment satisfaction across the nine sessions ranged from 6.01 to 6.45 (7 = highest satisfaction)
	Orsulic-Jeras [60], 2016	Dyads	Social and psychological intervention, Dyadic counseling-based, include support activities, and education.	The average satisfaction of dyads was 3.46 (5 = highest satisfaction)

### Acceptability of the interventions

#### Recruitment and participation rate

A total of 20 high- and moderate-quality papers presented the actual participation rate after recruitment. Twelve studies were rated as high (the actual participation rate was ≥ 80%), including 2 social [36, 54], 3 cognitive [48, 50, 61], one physical [45], and 6 other psychosocial interventions [25, 35, 43, 46, 55, 60]. Three multi-component psychosocial intervention studies were rated as moderate (60–79% participation) [17, 49, 52]. Five studies were rated as low (less than 60% participation), including one social [57], one physical [59], and 3 other psychosocial interventions [47, 64, 66].

One low quality study showed low participation rate (31.8%) after the recruitment [67].

#### Completion rate

Thirty-one high- and moderate-quality studies reported data on participants’ completion rate with the interventions, ranging from high to low levels. Sixteen papers showed high completion rate (80% participants completed the intervention), including 5 social [36, 37, 54, 57, 63], one cognitive [40], one physical [45] and 9 other psychosocial interventions [17, 44, 46, 49, 51, 53, 55, 56, 66]. Fourteen showed moderate completion rate (60–79% participants completed), including 4 cognitive [48, 50, 61, 62], one social [65], one physical [59] and 8 other psychosocial interventions [24, 25, 35, 47, 52, 58, 60, 64]. Only one cognitive intervention study showed low completion rate [38] (less than 60% participants completed the intervention).

Five low-quality papers reported data on participants’ completion rate for the interventions. Three studies showed moderate completion rate [34, 69, 70], while the other 2 reported low completion rate [67, 68].

#### Participants’ satisfaction with the interventions

Thirteen high- and moderate-quality studies reported either quantitative or qualitative data on participant satisfaction with the interventions. These data indicated overall good levels of satisfaction. Caregivers in two studies (one cognitive and one multi-component intervention) showed average satisfaction scores above 3 (5 = highest satisfaction) [38, 60], and another two American papers (one cognitive and one multi-component intervention) [25, 39] showed average satisfaction scores above 5 (7 = highest satisfaction). The caregivers said that they would be very likely to recommend the program to others [25]. In two Finland studies, including one self-management intervention and one nutritional intervention, most (more than 90%) of the dyads estimated that the intervention was useful, and 80% of dyads felt that they benefited from participation [41, 44]. Similarly, most of the participants (more than 90%) in three cognitive intervention studies and one social intervention study were satisfied with the program [43, 48, 57, 61]. A Spanish paper reported that 97.7% of caregivers and 88.6% of therapists considered the cognitive intervention to be useful, and 6 months later, 93.2% of caregivers and 86.3% of therapists still considered it to be useful [40]. Caregivers (73%) in another study who received a multi-component psychosocial intervention also indicated that they benefitted a great deal from participating in the project [42].

Three low-quality papers showed data about participant satisfaction. More than 80% of the dyads in 2 cognitive intervention studies (in Australia and America) said the training was perceived to be very useful [69, 71]. Caregivers (71%) in another study on multi-component intervention reported that there was nothing about the intervention that was unacceptable to them [34].

### Factors related to the acceptability of the interventions

#### Cognitive intervention

Caregivers in an individual cognitive stimulation therapy program [62] reported that it was difficult to fit in the intervention because of time constraints, such as having a full-time/part-time paid job. Pot [38] summarized that the primary reason for refusal of participation was a claimed lack of need. Liddle et al. [69] reported the influence of participants’ health status as barriers to intervention acceptability.

#### Social intervention

Caregivers highlighted the importance of appropriate content of the intervention. Mahoney’s [36] study reported that one of the most frequently used modules (the weekly caregiver conversation) was informative, making them feel more knowledgeable about dementia. Xiao et al. found that limited English proficiency and low literacy level in caregivers were identified as barriers to the program [37]. Another barrier was change of the intervention implementer, which was a main reason for caregivers who did not comply with the intervention [37].

#### Physical intervention

One study on physical intervention [59] noted anecdotally that caregiver burden was a barrier for acceptability, with many participants dropping out because of increased caregiver burden. Apart from that, some caregivers dropped from the study because of medical complications.

#### Other psychosocial interventions

Three studies [25, 41, 60] highlighted two key facilitators of acceptability: the professionalism of the intervention team and their friendly attitude. Two studies noted the importance of applicability of the intervention. Prick et al. [24] found that possibility to exercise during the intervention was the most named reason by the dyads to participate in the program. Approximately 68.5% of the dyads in the intervention group continued to exercise at home. Caregivers in a Resourcefulness Training program [26] said the intervention was too time-consuming, and they did not like the method for practicing the skills. Four studies reported participants’ health status as barriers to intervention acceptability. Prick et al. [24] found that physical complaints from the dyads were barriers for delivery of the exercise component. Similarly, some participants withdrew due to ill health in another 3 studies [25, 41, 64]. Woods and Joling et al. summarized that the primary reason for refusal of participation was a claimed lack of need for this intervention [64, 67].

## Discussion

### Key findings

This systematic review examined the acceptability of psychosocial interventions for dementia caregivers. Forty-two papers were included and showed some high- and moderate-quality evidence supporting the acceptability of psychosocial interventions, with important benefits yielded for caregivers. Currently, however, there is not adequate evidence from these studies indicating that the acceptability of psychosocial interventions for dementia caregivers has received enough attention from researchers. Most studies only included data about completion rate, and less than half of the papers reported on take-up rates and participants’ evaluation of interventions.

### Cognitive intervention

Ten studies (3 for dyads and 7 for caregivers only) on cognitive intervention were included, 3 reported high participation rate [48, 50, 61], 8 studies showed low to high completion rate [38, 40, 48, 50, 61, 62, 69, 70], 8 studies indicated good levels of satisfaction [38–40, 48, 61, 69, 71]. Three reported on barriers to acceptability, including time constraints [62], lack of need [38], participants’ poor health [69]. Completion rate in dyadic studies was more stable than caregiver-only studies, except that, no other discernable difference between dyadic studies or caregiver-only studies was noted. The included studies showed overall good levels of acceptability, while in another research, Milders et al. [73] indicated that the acceptability of cognitive intervention may be overlooked, responses from the participants gave an over optimistic impression of the acceptability. Therefore, acceptability of cognitive interventions is still to be explored in future studies.

### Social intervention

Six studies on social intervention were included. Three reported on participation rate [36, 54, 57], 6 studies showed moderate to high completion rate [36, 37, 54, 57, 63, 65], only one study reported caregivers’ satisfaction with the social intervention [57], and 2 reported on factors related to acceptability. Appropriate content of the intervention was a facilitator [36], while the change of implementers was a barrier [37], the evidence showed the importance of intervention content and stable implementation team. Apart from that, language and literacy problems in caregivers were identified as barriers to acceptability [37]. Similarly, Cook et al., in other studies, said that the diversity of languages and cultures acts as an obstacle to developing, testing and implementing evidence-based psychosocial interventions [19].

### Physical intervention

Two physical intervention studies were included, reporting both participation rate and completion rate [45, 59]. Caregiver burden and poor health status was reported as barriers to acceptability of exercise program [59]. Similarly, Lamotte et al. [74] indicated that caregiver burden and physical limitation were potential challenges of implementing exercise interventions, which could influence recruitment of participants and outcomes (such as caregiver burden or adherence to the exercise regimen). Considering the limited information included, acceptability of physical interventions is still to be explored in future studies.

### Other psychosocial intervention

Twenty-four studies included both social and psychological intervention as well as exercise elements, making it difficult to make a clear distinguishing between them. In addition, it was usually hard to clearly differentiate components between social interventions and psychological interventions [10], so we grouped these multi-component interventions studies together. Thirteen [17, 25, 35, 43, 46, 47, 49, 52, 55, 60, 64, 66, 67] of the 24 included studies reported on low to high participation rate, 20 [17, 24, 25, 34, 35, 44, 46, 47, 49, 51–53, 55, 56, 58, 60, 64, 66–68] showed low to high completion rate, 8 [17, 25, 34, 41–43, 56, 60] indicated participants’ satisfaction and 10 reported limited but important information about factors related to the acceptability of interventions. No discernable difference between dyadic studies or caregiver-only studies was noted.

One essentially important but usually ignored concept in most caregiving research is the implementation of intervention, which is highly related to acceptability of intervention. Acceptability often assumes the intervention was implemented as planned, which is not often the case [75]. Implementation fidelity is an important indicator for treatment outcome evaluation, as it truly reflects whether failure to replicate the expected outcomes is a problem with the intervention itself or of its application [76]. Besides, much of caregiving literature does not report data on treatment components, which make it difficult to evaluate exactly how the combination of different components contributed to the intervention’s success in multi-component interventions [11]. In the current review, only six included studies [25, 43, 46, 49, 56, 61] reported data on implementation measurement. Therefore, reporting data on implementation fidelity and treatment components are quiet important.

Additionally, when involved in an intervention program, caregivers were often required to make changes in their own caregiving behavior. Behavioral change is a process that unfolds over time, involving progression through six stages [77]. The stage of change model incorporate action as but one stage, preceded by precontemplation, contemplation, and preparation, and followed by maintenance and termination [78]. A series of studies reported that readiness to change was related to participants’ adherence to treatment [79, 80]. However, as a construct related to acceptability, participants’ readiness to change behavior have always been overlooked. In this review, only one included study [46] has assessed readiness to change among caregivers. They found that the stage of change was a predictive factor for adherence to the intervention. Examining readiness to change may yield valuable information about how to improve acceptability, and how to help participants fully benefit from interventions.

### Limitations

Several limitations of this review need to be considered. First, we reviewed both qualitative and quantitative studies, included a broad range of interventions, and used multiple outcomes and diverse measures of acceptability. The heterogeneity between these studies makes it impossible to meta-analyze the data or make comparisons between studies. Second, in an attempt to obtain the most comprehensive literature possible, we included some low-quality studies, and considering the limited number of high quality studies (only 50%). This may have resulted in potential bias in some areas. Finally, the implementation of psychosocial interventions is a complicated process that is dependent on the environment and where it occurred [81]. All the included studies came from developed countries, and 88% were delivered by specialists. Although we conducted a systematic search, the lack of data on developing countries and non-specialist delivered psychosocial interventions represents an important gap in the evidence. Additionally, data about participants’ evaluation of the interventions were mainly on satisfaction, and information on satisfaction was only provided for intervention completers. Limited information was available for caregivers who had discontinued with the intervention.

### Implications for future research and practice

The number of high-quality trials evaluating the effectiveness of psychosocial interventions for caregivers of people with dementia continues to increase [17, 82]. Those interventions, however, may be clinically effective but unacceptable to participants [83]. To fully understand and improve the acceptability of psychosocial interventions in practice, future studies need to evaluate the needs and readiness to change of caregivers, improve the applicability of interventions before implementation. Moreover, measures of acceptability of the interventions should be more comprehensive and should include drop-out rates, take-up rates and participants’ evaluations. To successfully translate effective interventions from research settings into real world practice, it is also important to make a practice manual that reports more details about implementation of the intervention, such as fidelity of implementation, attendance at sessions and how staff training for intervention implementers was undertaken.

As an indicator for acceptability, Satisfaction presumes the consumer can compare the current product (intervention) with other options. But caregivers do not necessarily know what else may be available. Therefore, satisfaction is an important indicator, because it indicates the experience was relatively positive, but the value is limited. Future studies should be more rigorous on evaluation of acceptability.

In addition, based on the result from this review, there is a lack of acceptability data on dementia caregivers in low- and middle-income countries. Future studies should also focus on the practicality and acceptability of psychosocial interventions in low- and middle-income countries, which are important and crucial to ease caregivers’ care burden and improve their quality of life [84].

## Conclusion

There is preliminary evidence to support the acceptability of psychosocial interventions for dementia caregivers. However, the available supporting evidence is limited, and there is currently no adequate information from these studies indicating that the acceptability has received enough attention from researchers. More well-designed studies assessing psychosocial interventions are needed to give specific statements about acceptability, and the measure of acceptability with psychosocial interventions should be more comprehensive.

## Additional files

Additional file 1: Search strategy. (DOCX 36 kb)

Additional file 2: Quality assessment. (DOCX 29 kb)

Additional file 3: Description and quality ratings of included studies. (DOCX 54 kb)

## Abbreviation

RCT: randomized control trial
