# Supplementary material for: Acceptability of psychosocial interventions for dementia caregivers: a systematic review
Source: BMC Psychiatry. 2019 Jan 14;19:23. doi: 10.1186/s12888-018-1976-4 (PMC6332684; doi:10.1186/s12888-018-1976-4)
Supplement: Supplementary file 1 — Search strategy. (DOCX 36 kb) [file 12888_2018_1976_MOESM1_ESM.docx]

**Search strategy (Main search: August, 2017)**

（web of science, Embase, Cochrane library, Pubmed, PsycARTICLES）

1. participants

1.

MeSH：dementia

“Alzheimer Disease”

“Frontotemporal Dementia”

“Vascular Dementia”

“Huntington Disease”

“Parkinson’s disease”

“Pick’s disease”

“Familial Dementia”

“Lewy body dementia”

2.

MeSH：“caregiver”

“caregiv*”

“care*”

“care giv*”

“family”

“family relations”

1 and 2

1. intervention

“intervention*”; “psychosocial intervention*”; “family intervention*”；“psycho-education*”; “education*”; “health education”; “Support group”; “mutual support group”; “social support”; “emotional support”; “information support”;；“social skills training”； “case management”; “cognitive rehabilitation”; “counseling intervention”; “learn*”; “train*”; “psychotherapy”; “treatment”； “program*”

1. Acceptability

“acceptability”; “feasibility”; “adherence”； “compliance”； “satisfaction”; “utilization”;

[1] and [2] and [3]

**Embase search strategy (Main search: August, 2017)**

Results Date

#47. (('dementia'/exp OR 'alzheimer disease':ti,ab OR 2,133 18 Aug 2017

'frontotemporal dementia':ti,ab OR 'vascular

dementia':ti,ab OR 'huntington disease':ti,ab OR

'parkinson disease':ti,ab OR 'pick presenile

dementia':ti,ab OR 'familial dementia':ti,ab OR

'lewy body dementia':ti,ab) AND ('caregiver'/exp

OR 'caregiv*':ti,ab OR 'care*':ti,ab OR 'care

giv*':ti,ab OR 'family':ti,ab OR 'family

relation':ti,ab)) AND ('intervention*':ti,ab OR

'psychosocial intervention*':ti,ab OR 'family

intervention*':ti,ab OR 'psycho-education*':ti,ab

OR 'education*':ti,ab OR 'health education':ti,ab

OR 'support group':ti,ab OR 'mutual support

group':ti,ab OR 'social support':ti,ab OR

'emotional support':ti,ab OR 'information

support':ti,ab OR 'social skills training':ti,ab

OR 'case management':ti,ab OR 'cognitive

rehabilitation':ti,ab OR 'counseling

intervention':ti,ab OR 'learn*':ti,ab OR

'train*':ti,ab OR 'psychotherapy':ti,ab OR

'treatment':ti,ab OR 'program*':ti,ab) AND

('feasibility':ti,ab OR 'acceptability':ti,ab OR

'adherence':ti,ab OR 'compliance':ti,ab OR

'satisfaction':ti,ab OR 'utilization':ti,ab)

#46. 'feasibility':ti,ab OR 'acceptability':ti,ab OR 783,540 18 Aug 2017

'adherence':ti,ab OR 'compliance':ti,ab OR

'satisfaction':ti,ab OR 'utilization':ti,ab

#45. 'utilization':ti,ab 192,873 18 Aug 2017

#44. 'satisfaction':ti,ab 141,258 18 Aug 2017

#43. 'compliance':ti,ab 147,404 18 Aug 2017

#42. 'adherence':ti,ab 130,538 18 Aug 2017

#41. 'acceptability':ti,ab 33,074 18 Aug 2017

#40. 'feasibility':ti,ab 187,656 18 Aug 2017

#39. 'intervention*':ti,ab OR 'psychosocial 7,062,406 18 Aug 2017

intervention*':ti,ab OR 'family

intervention*':ti,ab OR 'psycho-education*':ti,ab

OR 'education*':ti,ab OR 'health education':ti,ab

OR 'support group':ti,ab OR 'mutual support

group':ti,ab OR 'social support':ti,ab OR

'emotional support':ti,ab OR 'information

support':ti,ab OR 'social skills training':ti,ab

OR 'case management':ti,ab OR 'cognitive

rehabilitation':ti,ab OR 'counseling

intervention':ti,ab OR 'learn*':ti,ab OR

'train*':ti,ab OR 'psychotherapy':ti,ab OR

'treatment':ti,ab OR 'program*':ti,ab

#38. 'program*':ti,ab 915,948 18 Aug 2017

#37. 'treatment':ti,ab 4,887,264 18 Aug 2017

#36. 'psychotherapy':ti,ab 43,592 18 Aug 2017

#35. 'train*':ti,ab 550,535 18 Aug 2017

#34. 'learn*':ti,ab 383,091 18 Aug 2017

#33. 'counseling intervention':ti,ab 590 18 Aug 2017

#32. 'cognitive rehabilitation':ti,ab 1,853 18 Aug 2017

#31. 'case management':ti,ab 11,058 18 Aug 2017

#30. 'social skills training':ti,ab 1,190 18 Aug 2017

#29. 'information support':ti,ab 752 18 Aug 2017

#28. 'emotional support':ti,ab 6,140 18 Aug 2017

#27. 'social support':ti,ab 35,936 18 Aug 2017

#26. 'mutual support group':ti,ab 32 18 Aug 2017

#25. 'support group':ti,ab 4,453 18 Aug 2017

#24. 'health education':ti,ab 30,154 18 Aug 2017

#23. 'education*':ti,ab 569,414 18 Aug 2017

#22. 'psycho-education*':ti,ab 2,092 18 Aug 2017

#21. 'family intervention*':ti,ab 1,673 18 Aug 2017

#20. 'psychosocial intervention*':ti,ab 5,895 18 Aug 2017

#19. 'intervention*':ti,ab 1,019,742 18 Aug 2017

#18. ('dementia'/exp OR 'alzheimer disease':ti,ab OR 46,078 18 Aug 2017

'frontotemporal dementia':ti,ab OR 'vascular

dementia':ti,ab OR 'huntington disease':ti,ab OR

'parkinson disease':ti,ab OR 'pick presenile

dementia':ti,ab OR 'familial dementia':ti,ab OR

'lewy body dementia':ti,ab) AND ('caregiver'/exp

OR 'caregiv*':ti,ab OR 'care*':ti,ab OR 'care

giv*':ti,ab OR 'family':ti,ab OR 'family

relation':ti,ab)

#17. 'caregiver'/exp OR 'caregiv*':ti,ab OR 2,463,881 18 Aug 2017

'care*':ti,ab OR 'care giv*':ti,ab OR

'family':ti,ab OR 'family relation':ti,ab

#16. 'family relation':ti,ab 84 18 Aug 2017

#15. 'family':ti,ab 822,917 18 Aug 2017

#14. 'care giv*':ti,ab 7,510 18 Aug 2017

#13. 'care*':ti,ab 1,749,821 18 Aug 2017

#12. 'caregiv*':ti,ab 65,574 18 Aug 2017

#11. 'caregiver'/exp 58,186 18 Aug 2017

#10. 'dementia'/exp OR 'alzheimer disease':ti,ab OR 307,109 18 Aug 2017

'frontotemporal dementia':ti,ab OR 'vascular

dementia':ti,ab OR 'huntington disease':ti,ab OR

'parkinson disease':ti,ab OR 'pick presenile

dementia':ti,ab OR 'familial dementia':ti,ab OR

'lewy body dementia':ti,ab

#9. 'lewy body dementia':ti,ab 1,074 18 Aug 2017

#8. 'familial dementia':ti,ab 97 18 Aug 2017

#7. 'pick presenile dementia':ti,ab 1 18 Aug 2017

#6. 'parkinson disease':ti,ab 12,069 18 Aug 2017

#5. 'huntington disease':ti,ab 3,154 18 Aug 2017

#4. 'vascular dementia':ti,ab 7,770 18 Aug 2017

#3. 'frontotemporal dementia':ti,ab 7,644 18 Aug 2017

#2. 'alzheimer disease':ti,ab 18,187 18 Aug 2017

#1. 'dementia'/exp 294,127 18 Aug 2017

.......................................................

**Pubmed** **search strategy (Main search: August, 2017)**

Search ((((((((((((dementia[Title/Abstract]) OR Alzheimer Disease[Title/Abstract]) OR Frontotemporal Dementia[Title/Abstract]) OR Vascular Dementia[Title/Abstract]) OR Huntington Disease[Title/Abstract]) OR Parkinson’s disease[Title/Abstract]) OR Pick’s disease[Title/Abstract]) OR Lewy body dementia[Title/Abstract]) OR Familial Dementia[Title/Abstract])) AND ((((((caregiver[Title/Abstract]) OR caregiv*[Title/Abstract]) OR care*[Title/Abstract]) OR care giv*[Title/Abstract]) OR family[Title/Abstract]) OR family relations[Title/Abstract])) AND ((((((((((((((((((((((((intervention*[Title/Abstract]) OR psychosocial intervention*[Title/Abstract]) OR non-pharmacological intervention*[Title/Abstract]) OR psychological intervention*[Title/Abstract]) OR family intervention*[Title/Abstract]) OR psycho-education*[Title/Abstract]) OR education*[Title/Abstract]) OR health education[Title/Abstract]) OR support group[Title/Abstract]) OR mutual support group[Title/Abstract]) OR social support[Title/Abstract]) OR emotional support[Title/Abstract]) OR information support[Title/Abstract]) OR social skills training[Title/Abstract]) OR case management[Title/Abstract]) OR cognitive rehabilitation[Title/Abstract]) OR counseling intervention[Title/Abstract]) OR learn*[Title/Abstract]) OR train*[Title/Abstract]) OR psychotherapy[Title/Abstract]) OR treatment[Title/Abstract]) OR program*[Title/Abstract]) OR exercise[Title/Abstract]) OR physical activities[Title/Abstract])) AND ((((((acceptability[Title/Abstract]) OR feasibility[Title/Abstract]) OR adherence[Title/Abstract]) OR compliance[Title/Abstract]) OR satisfaction[Title/Abstract]) OR utilization[Title/Abstract])

**Search strategy (supplementary search: 02 June, 2018)**

**(1) participants**

1.

MeSH：dementia

“Alzheimer Disease”

“Frontotemporal Dementia”

“Vascular Dementia”

“Huntington Disease”

“Parkinson’s disease”

“Pick’s disease”

“Familial Dementia”

“Lewy body dementia”

2.

MeSH：“caregiver”

“caregiv*”

“care*”

“care giv*”

“family”

“family relations”

1. and 2

**(2) intervention**

'non-pharmacological intervention*'

' psycholog* intervention*'

' behavio* intervention*'

' physical '

' exercise '

' life style'

**(3) Acceptability**

“acceptability”; “feasibility”; “adherence”； “compliance”； “satisfaction”; “utilization”

**In total 2629 papers were identified:**

Pubmed: 501

Embase: 505

Web of science: 1153

Cohrane ：423

Psycarticle: 47

**Embase search strategy (supplementary search: 02 June, 2018)**

Embase：505

#32 #10 AND #17 AND #24 AND #31 505 02 June 2018

#31 #25 OR #26 OR #27 OR #28 OR #29 OR #30 846,416 02 June 2018

#30 'utilization':ti,ab 206,835 02 June 2018

#29 'satisfaction':ti,ab 153,759 02 June 2018

#28 'compliance':ti,ab 157,128 02 June 2018

#27 'adherence':ti,ab 142,930 02 June 2018

#26 'feasibility':ti,ab 204,673 02 June 2018

#25 'acceptability':ti,ab 36,352 02 June 2018

#24 #18 OR #19 OR #20 OR #21 OR #22 OR #23 1,013,874 02 June 2018

#23 'life style':ti,ab 15,131

#22 'exercise':ti,ab 300,210 02 June 2018

#21 'physical':ti,ab 755,711 02 June 2018

#20 'behavio* intervention*':ti,ab 10,898 02 June 2018

#19 'psycholog* intervention*':ti,ab 6,654 02 June 2018

#18 'non-pharmacological intervention*':ti,ab 2,173 02 June 2018

#17 #11 OR #12 OR #13 OR #14 OR #15 OR #16 2,620,338 02 June 2018

#16 'family relations':ti,ab 1,212 02 June 2018

#15 'family':ti,ab 869,443 02 June 2018

#14 'care giv*':ti,ab 8,005 02 June 2018

#13 'care*':ti,ab 1,875,824 02 June 2018

#12 'caregiv*':ti,ab 72,507 02 June 2018

#11 'caregiver':ti,ab 31,281 02 June 2018

#10 #1 OR #2 OR #3 OR #4 OR #5 OR #6 OR #7 OR #8 OR #9 158,867 02 June 2018

#9 'lewy body dementia':ti,ab 1,183 02 June 2018

#8 'familial dementia':ti,ab 98 02 June 2018

#7 'pick disease':ti,ab 2,189 02 June 2018

#6 'parkinson disease':ti,ab 12,977 02 June 2018

#5 'huntington disease':ti,ab 3,326 02 June 2018

#4 'vascular dementia':ti,ab 8,186 02 June 2018

#3'frontotemporal dementia':ti,ab 8,325 02 June 2018

#2 'alzheimer disease':ti,ab 19,188 02 June 2018

#1 dementia:ti,ab 129,955 02 June 2018

**Pubmed search strategy (supplementary search: 02 June, 2018)**

(((((dementia[MeSH Terms]) OR ((((((((Alzheimer Disease[Title/Abstract]) OR Frontotemporal Dementia[Title/Abstract]) OR Vascular Dementia[Title/Abstract]) OR Huntington Disease[Title/Abstract]) OR Parkinson’s disease[Title/Abstract]) OR Pick’s disease[Title/Abstract]) OR Familial Dementia[Title/Abstract]) OR Lewy body dementia[Title/Abstract]))) AND ((caregiver[MeSH Terms]) OR (((((caregiv*[Title/Abstract]) OR care*[Title/Abstract]) OR care giv*[Title/Abstract]) OR family[Title/Abstract]) OR family relations[Title/Abstract]))) AND ((((((non-pharmacological intervention*[Title/Abstract]) OR physical[Title/Abstract]) OR psycholog* intervention*[Title/Abstract]) OR behavio* intervention*[Title/Abstract]) OR exercise[Title/Abstract]) OR life style[Title/Abstract])) AND ((((((acceptability[Title/Abstract]) OR feasibility[Title/Abstract]) OR adherence[Title/Abstract]) OR compliance[Title/Abstract]) OR satisfaction[Title/Abstract]) OR utilization[Title/Abstract])

**Search strategy (updata search: from 17 August, 2017 to 02 June, 2018)**

（web of science, Embase, Cochrane library, Pubmed, PsycARTICLES）

1. participants

1.

MeSH：dementia

“Alzheimer Disease”

“Frontotemporal Dementia”

“Vascular Dementia”

“Huntington Disease”

“Parkinson’s disease”

“Pick’s disease”

“Familial Dementia”

“Lewy body dementia”

2.

MeSH：“caregiver”

“caregiv*”

“care*”

“care giv*”

“family”

“family relations”

1 and 2

1. intervention

“intervention*”; “psychosocial intervention*”; “family intervention*”；“psycho-education*”; “education*”; “health education”; “Support group”; “mutual support group”; “social support”; “emotional support”; “information support”;；“social skills training”； “case management”; “cognitive rehabilitation”; “counseling intervention”; “learn*”; “train*”; “psychotherapy”; “treatment”； “program*”

1. Acceptability

“acceptability”; “feasibility”; “adherence”； “compliance”； “satisfaction”; “utilization”;

[1] and [2] and [3]

**In total 733 papers were identified:**

Pubmed: 50

Embase: 276

Web of science: 270

The Cohrane Library：125

Psycarticle: 12

**Embase search strategy (updata search: from 17 August, 2017 to 02 June, 2018)**

**Embase: 276**

#47 #18 AND #39 AND #46 276

#46 #40 OR #41 OR #42 OR #43 OR #44 OR # 45 68,150

#45 'utilization':ti,ab AND [17-8-2017]/sd NOT [2-6-2018]/sd 15,085

#44 'satisfaction':ti,ab AND [17-8-2017]/sd NOT [2-6-2018]/sd 13,587

#43 'compliance':ti,ab AND [17-8-2017]/sd NOT [2-6-2018]/sd 10,477

#42 'adherence':ti,ab AND [17-8-2017]/sd NOT [2-6-2018]/sd 13,452

#41 'feasibility':ti,ab AND [17-8-2017]/sd NOT [2-6-2018]/sd 18,459

#40 'acceptability':ti,ab AND [17-8-2017]/sd NOT [2-6-2018]/sd 3,574

#39 #19 OR #20 OR #21 OR #22 OR #23 OR #24 OR #25 OR #26 OR #27 OR #28 OR #29 OR #30 OR #31 OR #32 OR #33 OR #34 OR #35 OR #36 OR #37 OR #38 490,530

#38 'program*':ti,ab AND [17-8-2017]/sd NOT [2-6-2018]/sd 65,868

#37 'treatment':ti,ab AND [17-8-2017]/sd NOT [2-6-2018]/sd 325,205

#36 'psychotherapy':ti,ab AND [17-8-2017]/sd NOT [2-6-2018]/sd 1,576

#35 'train*':ti,ab AND [17-8-2017]/sd NOT [2-6-2018]/sd 43,864

#34 'learn*':ti,ab AND [17-8-2017]/sd NOT [2-6-2018]/sd 34,434

#33 'counseling intervention':ti,ab AND [17-8-2017]/sd NOT [2-6-2018]/sd 35

#32 'cognitive rehabilitation':ti,ab AND [17-8-2017]/sd NOT [2-6-2018]/sd 178

#31 'case management':ti,ab AND [17-8-2017]/sd NOT [2-6-2018]/sd 740

#30 'social skills training':ti,ab AND [17-8-2017]/sd NOT [3-6-2018]/sd 70

#29 'information support':ti,ab AND [17-8-2017]/sd NOT [2-6-2018]/sd 54

#28 'emotional support':ti,ab AND [17-8-2017]/sd NOT [2-6-2018]/sd 551

#27 'social support':ti,ab AND [17-8-2017]/sd NOT [2-6-2018]/sd 3,087

#26 'mutual support group':ti,ab AND [17-8-2017]/sd NOT [2-6-2018]/sd 2

#25 'support group':ti,ab AND [17-8-2017]/sd NOT [2-6-2018]/sd 355

#24 'health education':ti,ab AND [17-8-2017]/sd NOT [2-6-2018]/sd 1,751

#23 'education*':ti,ab AND [17-8-2017]/sd NOT [2-6-2018]/sd 42,966

#22 'psycho-education*':ti,ab AND [17-8-2017]/sd NOT [2-6-2018]/sd 166

#21 'family intervention*':ti,ab AND [17-8-2017]/sd NOT [2-6-2018]/sd 104

#20 'psychosocial intervention*':ti,ab AND [17-8-2017]/sd NOT [2-6-2018]/sd 565

#19 'intervention*':ti,ab AND [17-8-2017]/sd NOT [2-6-2018]/sd 101,718

#18 #10 AND #17 3,212

#17 #11 OR #12 OR #13 OR #14 OR #15 OR #16 177,675

#16'family relations':ti,ab AND [17-8-2017]/sd NOT [2-6-2018]/sd 56

#15'family':ti,ab AND [17-8-2017]/sd NOT [2-6-2018]/sd 49,736

#14 'care giv*':ti,ab AND [17-8-2017]/sd NOT [2-6-2018]/sd 527

#13 'care*':ti,ab AND [17-8-2017]/sd NOT [2-6-2018]/sd 138,091

#12 'caregiv*':ti,ab AND [17-8-2017]/sd NOT [2-6-2018]/sd 7,447

#11 'caregiver':ti,ab AND [17-8-2017]/sd NOT [2-6-2018]/sd 3,325

#10 #1 OR #2 OR #3 OR #4 OR #5 OR #6 OR #7 OR #8 OR #9 11,592

#9 'lewy body dementia':ti,ab AND [17-8-2017]/sd NOT [2-6-2018]/sd 114

#8 'familial dementia':ti,ab AND [17-8-2017]/sd NOT [2-6-2018]/sd 1

#7 'pick disease':ti,ab AND [17-8-2017]/sd NOT [2-6-2018]/sd 93

#6 'parkinson disease':ti,ab AND [17-8-2017]/sd NOT [2-6-2018]/sd 981

#5 'huntington disease':ti,ab AND [17-8-2017]/sd NOT [2-6-2018]/sd 188

#4 'vascular dementia':ti,ab AND [17-8-2017]/sd NOT [2-6-2018]/sd 464

#3 'frontotemporal dementia':ti,ab AND [17-8-2017]/sd NOT [2-6-2018]/sd 748

#2 'alzheimer disease':ti,ab AND [17-8-2017]/sd NOT [2-6-2018]/sd 1,115

#1 dementia:ti,ab AND [17-8-2017]/sd NOT [2-6-2018]/sd 9,793

**Pubmed** **search strategy (updata search: August, 2017)**

Search ((((((((((((dementia[Title/Abstract]) OR Alzheimer Disease[Title/Abstract]) OR Frontotemporal Dementia[Title/Abstract]) OR Vascular Dementia[Title/Abstract]) OR Huntington Disease[Title/Abstract]) OR Parkinson’s disease[Title/Abstract]) OR Pick’s disease[Title/Abstract]) OR Lewy body dementia[Title/Abstract]) OR Familial Dementia[Title/Abstract])) AND ((((((caregiver[Title/Abstract]) OR caregiv*[Title/Abstract]) OR care*[Title/Abstract]) OR care giv*[Title/Abstract]) OR family[Title/Abstract]) OR family relations[Title/Abstract])) AND ((((((((((((((((((((((((intervention*[Title/Abstract]) OR psychosocial intervention*[Title/Abstract]) OR non-pharmacological intervention*[Title/Abstract]) OR psychological intervention*[Title/Abstract]) OR family intervention*[Title/Abstract]) OR psycho-education*[Title/Abstract]) OR education*[Title/Abstract]) OR health education[Title/Abstract]) OR support group[Title/Abstract]) OR mutual support group[Title/Abstract]) OR social support[Title/Abstract]) OR emotional support[Title/Abstract]) OR information support[Title/Abstract]) OR social skills training[Title/Abstract]) OR case management[Title/Abstract]) OR cognitive rehabilitation[Title/Abstract]) OR counseling intervention[Title/Abstract]) OR learn*[Title/Abstract]) OR train*[Title/Abstract]) OR psychotherapy[Title/Abstract]) OR treatment[Title/Abstract]) OR program*[Title/Abstract]) OR exercise[Title/Abstract]) OR physical activities[Title/Abstract])) AND ((((((acceptability[Title/Abstract]) OR feasibility[Title/Abstract]) OR adherence[Title/Abstract]) OR compliance[Title/Abstract]) OR satisfaction[Title/Abstract]) OR utilization[Title/Abstract])
