# Supplementary material for: Acceptability of psychosocial interventions for dementia caregivers: a systematic review
Source: BMC Psychiatry. 2019 Jan 14;19:23. doi: 10.1186/s12888-018-1976-4 (PMC6332684; doi:10.1186/s12888-018-1976-4)
Supplement: Supplementary file 2 — Quality assessment. (DOCX 29 kb) [file 12888_2018_1976_MOESM2_ESM.docx]

**Quality assessment**

**1. Quantitative table checklist ( 1 = STRONG, 2 = MODERATE, 3 = WEAK)**

| **Article** | **Selection bias** | **study design** | **Confounders addressed** | **Blinding** | **data collection tools** | **Withdrawals and dropouts** | **Intervention integrity** | **appropriate**  **analysis** | **Value of research discussed** | **Assessment** |
| --- | --- | --- | --- | --- | --- | --- | --- | --- | --- | --- |
| USA  Tremont [17], 2015 | 2 | 1 | 1 | 2 | 1 | 1 | 1 | 1 | 1 | 1 |
| USA  Winter [64], 2007 | 2 | 1 | 2 | 2 | 1 | 1 | 2 | 2 | 2 | 2 |
| Netherlands  Prick [24],  2014 | 2 | 1 | 3 | 1 | NA | 2 | 2 | 1 | 2 | 2 |
| America  Mahoney [36], 2001. | 1 | 1 | 1 | 2 | 2 | 1 | 1 | 2 | 1 | 1 |
| Australia  Xiao [37], 2015 | 2 | 1 | 2 | 2 | 1 | 1 | 1 | 2 | 1 | 1 |
| Netherlands  Jansen [56], 2011 | 3 | 1 | 1 | 1 | 1 | 1 | 2 | 1 | 1 | 2 |
| Netherlands  Joling [68], 2013 | 3 | 1 | 2 | 2 | 1 | 3 | 2 | 1 | 1 | 3 |
| USA  Beauchamp [39], 2005 | 1 | 1 | 1 | 2 | 1 | 2 | 2 | 1 | 1 | 1 |
| Netherlands  Pot [38], 2015 | 1 | 1 | 2 | 2 | 1 | 2 | 2 | 1 | 1 | 1 |
| USA  Hebert [57],1994 | 3 | 1 | 2 | 2 | 1 | 1 | 1 | 2 | 1 | 2 |
| USA  Czaja [42], 2013 | 1 | 1 | 2 | 2 | 1 | 2 | 1 | 1 | 1 | 1 |
| USA  Coon [58], 2003 | 2 | 1 | 3 | 2 | 1 | 2 | 1 | 2 | 1 | 2 |
| USA  Castro [59],2002 | 3 | 1 | 1 | 2 | 1 | 1 | 1 | 1 | 2 | 2 |
| USA  Whitlatch [25], 2006 | 2 | 2 | 3 | 2 | 1 | 2 | 2 | 1 | 1 | 2 |
| Sweden  Dahlrup, B. [69], 2014 | 3 | 2 | 3 | 2 | 1 | 1 | 3 | 1 | 1 | 3 |
| USA  Orsulic-Jeras [60], 2016 | 2 | 2 | 3 | 2 | 1 | 2 | 1 | 2 | 1 | 2 |
| USA  McCurry [61], 2015 | 1 | 2 | 3 | 2 | 1 | 2 | 1 | 2 | 2 | 2 |
| Spain  Martin-Carrasco [40], 2009 | 1 | 1 | 1 | 2 | 1 | 1 | 1 | 1 | 1 | 1 |
| Australia  Liddle [70], 2012 | 3 | 2 | 3 | 2 | 1 | 1 | 1 | 2 | 1 | 3 |
| USA  Piercy [63], 2011 | 2 | 1 | 1 | 3 | 1 | 2 | 2 | 2 | 1 | 2 |
| USA  Chee [46], 2007 | 1 | 1 | 1 | 2 | 1 | 2 | 1 | 1 | 1 | 1 |
| USA  Callan [71], 2015 | 3 | 1 | 3 | 2 | 1 | 3 | 2 | 2 | 2 | 3 |
| USA  Zauszniewski [26], 2016 | 3 | 2 | 2 | 2 | 1 | 1 | 1 | 2 | 1 | 2 |
| USA  Gaugler [72], 2015 | 3 | 2 | 3 | 2 | 2 | 3 | 1 | 2 | 1 | 3 |
| UK  Woods [65], 2016 | 1 | 1 | 1 | 2 | 1 | 3 | 1 | 1 | 1 | 2 |
| Finland  Laakkonen [44], 2013 | 2 | 1 | 2 | 1 | 1 | 2 | 2 | 1 | 2 | 1 |
| Finland  Pitkala [45], 2011 | 2 | 1 | 2 | 2 | 1 | 1 | 2 | 2 | 1 | 1 |
| UK  Livingston [47],  2014 | 2 | 1 | 1 | 2 | 1 | 2 | 1 | 1 | 1 | 1 |
| Germany  Wilz [48],  2016 | 1 | 1 | 2 | 2 | 1 | 2 | 1 | 1 | 1 | 1 |
| USA  Burgio [49],  2003 | 2 | 1 | 2 | 2 | 1 | 1 | 2 | 1 | 1 | 1 |
| France  Rotrou [50],  2011 | 1 | 1 | 2 | 2 | 1 | 2 | 1 | 1 | 1 | 1 |
| Canada  Mohide [66],  1990 | 3 | 2 | 2 | 2 | 1 | 2 | 2 | 2 | 1 | 2 |
| Europe  Barbabella [35],  2018 | 3 | 2 | 2 | 2 | 1 | 2 | 2 | 1 | 1 | 2 |
| USA  Roberts [34],2009 | 3 | 3 | 2 | 2 | 1 | 2 | 2 | 1 | 1 | 3 |
| Denmark  Søgaard [51],  2014 | 2 | 1 | 2 | 2 | 1 | 1 | 2 | 1 | 1 | 1 |
| USA  Belle [52],  2006 | 2 | 1 | 1 | 2 | 1 | 2 | 1 | 1 | 1 | 1 |
| Hong Kong  Kwok [53],  2012 | 2 | 1 | 2 | 2 | 1 | 1 | 2 | 1 | 1 | 1 |
| USA  Vickrey [67],  2006 | 3 | 1 | 2 | 2 | 1 | 1 | 2 | 2 | 1 | 2 |
| Finland  Eloniemi-Sulkava [54],  2009 | 2 | 1 | 2 | 2 | 1 | 1 | 1 | 1 | 1 | 1 |
| USA  Mittelman [55],  2006 | 1 | 1 | 2 | 2 | 1 | 1 | 1 | 1 | 1 | 1 |

**2. Qualitative data study checklist (Y=Yes, N=No, I=Insufficient information) (STRONG, ADEQUATE, WEAK, UNKNOWN)**

| **Article(s)** | **Clear aims** | **Appropriate methodology** | **Appropriate research design** | **Detailed, justified, recruitment strategy** | **Appropriate data collection methods** | **Researcher-participant relationship considered** | **Ethical issues considered** | **Rigorous data analysis** | **Clear findings** | **Value of research discussed** | **Assessment** |
| --- | --- | --- | --- | --- | --- | --- | --- | --- | --- | --- | --- |
| Finland  Puranen [41], 2014 | **Y** | **Y** | **Y** | **Y** | **Y** | **N** | **Y** | **Y** | **Y** | **Y** | **STRONG** |
| USA  Zarit [43], 2013. | **Y** | **Y** | **Y** | **I** | **Y** | **N** | **Y** | **Y** | **Y** | **Y** | **STRONG** |
| UK  Leung [62], 2017 | **Y** | **Y** | **Y** | **I** | **Y** | **N** | **N** | **Y** | **Y** | **Y** | **ADEQUATE** |
| USA  Zauszniewski [26], 2016 | **Y** | **N** | **I** | **I** | **Y** | **I** | **I** | **Y** | **Y** | **Y** | **ADEQUATE** |
| Europe  Barbabella [35],  2018 | **Y** | **Y** | **I** | **I** | **Y** | **N** | **I** | **Y** | **Y** | **Y** | **ADEQUATE** |
| USA  Roberts [34],2009 | **Y** | **Y** | **Y** | **N** | **Y** | **N** | **N** | **Y** | **Y** | **Y** | **WEAK** |
