# Supplementary material for: Acceptability of psychosocial interventions for dementia caregivers: a systematic review
Source: BMC Psychiatry. 2019 Jan 14;19:23. doi: 10.1186/s12888-018-1976-4 (PMC6332684; doi:10.1186/s12888-018-1976-4)
Supplement: Supplementary file 3 — Description and quality ratings of included studies. (DOCX 54 kb) [file 12888_2018_1976_MOESM3_ESM.docx]

**Table 2 Description and quality ratings of included studies**

| **Author** | **Study design** | **Sample** | **Intervention** | **Intervention workforce** | **Setting** | **Measures of acceptability identified** | **Quality assessment** |
| --- | --- | --- | --- | --- | --- | --- | --- |
| USA  Tremont [17], 2015 | RCT | 250 caregivers;  intervention group (n=133);  control group (n=117) | **Intervention group:**  **Social and psychological intervention** (6 months with 16 calls, include an 1-hour call, 6 weekly calls, 9 calls every two weeks)  Include: dementia education, emotional support, directing caregivers to appropriate resources, encouraging caregivers to attend to their physical, emotional, and social needs, coping strategies  **Control group:** nondirective support for caregivers through empathic and reflective listening and open-ended questioning. | Therapist | Academic medical center | **Acceptability of psychosocial interventions:**  **1) Recruitment and participation rate**  screened 477 individuals, 103 were not meeting inclusion/exclusion criteria, enrolled 250.  **2) Dropout analysis**  Fifteen percent of participants dropped from the study by 6-month follow up  **3) Satisfaction survey**  Overall satisfaction rates (1=not satisfied to 4=very much satisfied) did not significantly differ between the groups (FITT-C M=3.83, SD=0.51). | **Strong** |
| USA  Winter [63], 2006 | RCT | 103 female caregivers | **Intervention group:**  **Social Support group intervention (**telephone-based/6 months, one hour per week **)**  providing emotional support, coping strategies  **Control group:** usual care | Social workers | Telephone-based | **Acceptability of psychosocial interventions:**  **Completion rate**  Ninety-four caregivers (91.3%) were available for the 6-month telephone interview | **Moderate** |
| Finland  Puranen [41], 2014 | Qualitative study | 99 dyads;  intervention group (50);  control group (49) | **Intervention group:**  **Nutritional intervention**(12 months)  1)tailored nutritional advice on the basis of the food diaries  2)home visit 4-8 times  3)discussions with participants and weight control every third month  **Control group:**  received a written guide on nutrition | Nutritionist | Home | **Acceptability of psychosocial interventions:**  **Satisfaction** **survey**  Most (93%) of the participants estimated that the nutritionist’s home visits and guidance were useful for them, while 4% found that visits to be somewhat useful  Most (80%) of the participants felt that they benefited from their participation  **Factors related to acceptability:**  **Facilitators of acceptability**, professionalism of the intervention team  **Barriers to acceptability**, physical complaints | **Strong** |
| Netherlands  Prick [24], 2014 | RCT | 111 dyads;  intervention group (n=57);  comparison group (n=54) | **Intervention group:**  **multi-component psychosocial intervention(**3 months/8 one-hour sessions**)**  physical exercise training and support existing of psycho-education, communication skills training, pleasant activities training  **Control group:** Routine medical care | Personal coach | Home | **Acceptability of psychosocial interventions:**  **Completion rate**  44 dyads (77.2%) completed all eight home visits  **Factors related to acceptability:**  **Facilitators of acceptability**, applicability of the intervention; **Barriers to acceptability**, physical complaints | **Moderate** |
| USA  Mahoney [36], 2001 | RCT | 100 caregivers;  intervention group (n=49);  control group (n=51) | **Intervention group**:  **Social support (**12 months/ telephone-based**)**  Monitoring and counseling, In-home support group, Expert support, Activity/caregiver respite conversation  **Control group**:  usual care services and were offered use of the system after the study period | Research assistant (not specified) | telephone-based | **Acceptability of psychosocial interventions:**  **1) Recruitment and participation rate**  Of the 143 referrals from the recruitment sites, 118 were eligible and a total of 100 caregivers were enrolled  2) **Completion rate**  Two participants in the control group and one in the intervention group dropped out during the study  **Factors related to the acceptability:**  **Facilitators of acceptability**, appropriate content of the intervention | **Strong** |
| Australia  Xiao [37], 2015 | RCT | 72 caregivers;  intervention group (n=40);  control group (n=32) | **Intervention group:**  **Social support**  Personalized Dementia Care Intervention (12 months/one call per month)  Based on caregivers’ need, provide face-to-face coaching and support  **Control group:** Usual care | Care coordinators (a nurse, a social worker, and 6 Community Home Care Certificate holders) | Home | **Acceptability of psychosocial interventions:**  **1)** **Completion rate**  61 of them completed the trial at 12 months  **2) Satisfaction survey**  The caregivers in the intervention group demonstrated a significant increase in satisfaction with services compared with the usual care group  **Factors related to the acceptability:**  **Barriers to acceptability**, limited English proficiency and low literacy level; the change of intervention implementers. | **Strong** |
| Netherlands  Jansen [56], 2011 | RCT | 99 dyads;  intervention group (n=54);  control group (n=45) | **Intervention group:**  **Case management(**12 months/ met monthly**)**  2 home visits, formulate care plan, family-meeting,  **Control group:** Usual care depending on their own initiative. | Nurse | Home | **Acceptability of psychosocial interventions:**  **1) Completion rate**  81 participating pairs were retained in the trial at 12 months  **2) Caregiver-completed questionnaire**  Overall, caregivers were satisfied with the quality of the intervention. | **Moderate** |
| Netherlands  Joling [67], 2013 | RCT | 192 dyads;  intervention group (n=96);  control group (n=96) | **Intervention group:**  **Social and Psychological intervention(**12 months**/**six in-person counseling sessions/every 2 to 3 months**)**  psycho-education, teach problem solving techniques and mobilize the existing family networks, improve emotional and instrumental support.  **Control group:** usual care, free  to use all types of care. | Counselors | Home | **Acceptability of psychosocial interventions:**  **1) Recruitment and participation rate**  683 enrolled, 410 refused to participant, 81 not meeting the inclusion criteria, 192 attended.  **2) Completion rate**  44 (46%) intervention and 57 (59%) usual care group caregivers.  **Factors related to acceptability:**  **Barriers to acceptability**, lack of need | **Weak** |
| Netherlands  Pot [38], 2015 | RCT | 245 caregivers;  intervention group (n=149);  control group (n=96) | **Intervention group:**  **Cognitive intervention (**2 months/one lesson per week**)**  Psycho-education, include Problem-solving, Behavioral activation, Time-management, Cognitive restructuring.  **Control group:** minimal intervention | Psychologist | Internet-based | **Acceptability of psychosocial interventions:**  **1) Completion rate**  68 caregivers (45.6%)  completed all lessons within six months  **2) Satisfaction survey**  the mean score for usefulness was 4.16, whereas the comprehensibility had a mean score of 3.98  **Factors related to acceptability:**  **Barriers to acceptability**, lack of need | **Strong** |
| USA  Beauchamp [39], 2005 | RCT | 299 caregivers;  intervention group (n=150);  control group (n=149) | **Intervention group:**  **cognitive intervention (**1 month**/** Internet-based**)**  Include knowledge, cognitive, and behavioral skills training.  **Control group:** waitlist control | Not reported | Internet-based | **Acceptability of psychosocial interventions:**  **Satisfaction Survey**  Treatment participants indicated that they were quite satisfied with the program (M=5.1; SD=1.2); found the information to be quite useful (M=5.0, SD=1.3); indicated that the program was quite enjoyable (M=4.6; SD=1.3) | **Strong** |
| USA  Hebert [57], 1994 | RCT | 41 caregivers;  intervention group (n=23);  control group (n=18) | **Intervention group:**  **Support group intervention (**2 months/1-hour session per week**)**  Dementia education, behaviour or emotional Problem-solving, relaxation techniques-learning.  **Control group:** informal monthly meeting | Nurse | Not reported | **Acceptability of psychosocial interventions:**  **1) Recruitment and participation rate**  A total of 121 subjects were referred for participation in the study. Of these, 116 were reached and 85 fulfilled the eligibility criteria. Only 45 agreed to participate and signed the consent form  **2) Completion rate**  Four subjects dropped out before the first assessment  **3) Satisfaction Survey**  The majority of participants (95.9%) were satisfied or very satisfied with the Program and found it useful or very useful (95%) in their daily living. | **Moderate** |
| USA  Czaja [42], 2013 | RCT | 110 caregivers;  intervention group (n=38);  attention control group (n=36);  Information-only control group (n=36) | **Intervention group:**  **multi-component psychosocial intervention(**technology-based/5 months/6 1-hour monthly sessions**)**  problem-solving strategies, stress management, communication strategies, healthy behavior strategies  **Attention control group:** nutrition and healthy eating strategies  **Control group:** basic information about dementia and Alzheimer’s disease, caregiving | Certified interventionists | Home | **Acceptability of psychosocial interventions:**  **Satisfaction Survey**  The majority of caregivers (73%) receiving the intervention indicated that they benefitted a great deal from participating in the project  Most (82%) found the Video care network helpful and that the videophone was easy to use (85%). | **Strong** |
| USA  Coon [58], 2003 | RCT | 169 caregivers;  anger management group (n=41);  depression management group (n=45);  waiting list group (n=44) | **Intervention group:**  **Psychological intervention(**3 months/10 2-hour sessions**)**  Include anger management class and depression management class  **Control group:** waitlist control | Trained facilitators (psychologist/clinical interns/graduate student) | Not reported | **Acceptability of psychosocial interventions:**  **Completion rate**  39 (23%) dropped out of the study before completing their program | **Moderate** |
| USA  Castro [59], 2002 | RCT | 100 caregivers  intervention group (n=51);  control group (n=49) | **Intervention group:**  **Exercise intervention**(12 months/4 sessions per week)  exercise program  **Control group:** nutrition education | Health educators | Home | **Acceptability of psychosocial interventions:**  **1) Recruitment and participation rate**  A total of 574 women were initially screened for eligibility, 331 were excluded on the basis of study criteria, 143 caregivers were no longer interested in participating, 100 agreed to participate.  **2) Completion rate**  Participants achieved an average adherence rate of 74% with an average of 35 minutes per session.  **Factors related to acceptability:**  **Barriers to acceptability**, increased caregiver burden; medical complications. | **Moderate** |
| USA  Roberts [34], 2009 | quasi-experiment | 37 dyads | **Social and psychological intervention(**1 month /one session per week**)**  individual and family consultations, support group, weekly four-session education and support program | Trained facilitator with experience in dementia care | Community councils on aging | **Acceptability of psychosocial interventions:**  **1) Completion rate**  74% across 9 sessions  **2) Satisfaction Questionnaire**  71% gave it a letter grade of “A,” 99% found it helpful | **Weak** |
| USA  Zarit [43], 2013 | Exploratory Study | 35 caregivers | **Social and psychological intervention**  Advanced Caregiver Education and Support Program | Counselors | Home | **Acceptability of psychosocial interventions:**  **1) Participation rate**  90% agreed to participate the study  **2) Satisfaction scale**  Overall satisfaction with the program was high with 66% of caregivers indicating they were very satisfied, and 28% reporting they were somewhat satisfied. | **Strong** |
| USA  Whitlatch [25], 2006 | quasi-experiment | 31 dyads | **EDDI dyadic program** (9 sessions/60-100 min per session)  Address emotionally sensitive Issues, communication skills, Dementia education | Counselors | Home | **Acceptability of psychosocial interventions:**  **1) Recruitment and participation rate**  Recruited and enrolled 34 dyads in the EDDI program. Of these, 3 decided not to participate  **2) Completion rate**  65% dyads completed all sessions  **3) Satisfaction survey**  Caregiver ratings of treatment satisfaction across the nine sessions ranged from 6.01 to 6.45 out of a possible 7 points (SD=0.63–0.80);  **Factors related to acceptability:**  **Facilitators of acceptability**, professionalism of the intervention team; **Barriers to acceptability**, physical complaints | **Moderate** |
| Sweden  Dahlrup, B [68], 2014 | quasi-experiment and part of a longitudinal cohort study | 308 family caregivers; intervention group (n=153);  control group (n=155) | **social and psychological intervention (5 weeks/**2 hours per week/5 years follow-up**)**  include dementia education, support group | Counselors | Social setting | **Acceptability of psychosocial interventions:**  **Completion rate**  The drop-out rate was 11% in the intervention group and 19% among the controls, only 2 or 4 percentages remained in the study during the total follow-up of 60 months | **Weak** |
| USA  Orsulic-Jeras [60], 2016 | quasi-experiment | 40 dyads | **Dyadic counseling-based care-planning intervention**(7 sessions)  include support, activities, and education | Counselors | Not reported | **Acceptability of psychosocial interventions:**  **1) Recruitment and participation rate**  49 dyads were enrolled in the study, 9 refused  **2) Completion rate**  65% dyads completed all sessions  **3) Satisfaction Questionnaire**  Ratings on the 5-point scale indicated a high level of satisfaction for both the CGs (M=3.46; SD=.76) and PWDs (M=3.65; SD=.60)  **Factors related to acceptability:**  **Facilitators of acceptability**, professionalism of the intervention team | **Moderate** |
| USA  McCurry [61], 2015 | quasi-experimental study | 151 dyads | **Cognitive intervention** (2 months/one session per week)  Include Problem-solving skills, Effective communication skills, negative thinking management, Coping strategies, education. | Staff from regional Area Agencies on Aging | Home | **Acceptability of psychosocial interventions:**  **1) Recruitment and participation rate**  189 dyads were screened, 158 were eligible to participate, 151 chose to enter treatment  **2) Completion rate**  64% caregivers completed the program  **3) Satisfaction Questionnaire**  92% of caregivers who returned the survey (n = 79) felt the program had been very or somewhat helpful, and 80% said they would definitely or probably continue to use STAR-C skills. | **Moderate** |
| Spain  Martin-Carrasco [40], 2009 | multicenter, prospective, randomized study  RCT | 115 caregivers;  intervention group (n=60);  control group (n=55) | **Intervention group:**  **Cognitive Psycho-educational intervention** (4 months/ 8 90-min sessions/per 1-2 week)  Behavioural problems handing strategies, tension and stress control, dementia education.  **Control group:** standard care | Psychiatrist and therapist | Hospital and non-hospital psychiatric outpatient clinics | **Acceptability of psychosocial interventions:**  **1) Completion rate**  104 (90.4%) of them completed the study  **2) Satisfaction survey**  caregivers and therapists was generally positive, considering the majority of the caregivers that program was ‘useful’ or ‘very useful’ once the PIP had finished (97.7%), and 6 months later (93.2%), while 88.6% of the therapists considered it to be ‘useful’ or ‘very useful’ once the PIP had finished and 86.3%, 6 months later. | **Strong** |
| Australia  Liddle [69], 2012 | pre-test/post-test controlled trial | 29 dyads;  intervention group (n=13);  control group (n=16) | **Intervention group:**  **Cognitive intervention (**two 45-minute sessions**)**  Memory and communication training support;  **Control group:** the training DVD was given to them after the follow-up assessment was completed | Psychologist/speech pathologist | Home | **Acceptability of psychosocial interventions:**  **1) Completion rate**  7 dyads drop out  **2) Satisfaction survey**  the training was perceived to be very useful (85%, n=11) or fairly useful (15%, n=2). At follow-up, participants continued to feel positive about the program: 100% (n=13) of the group indicated it was fairly or very helpful, and all had found it easy or moderately easy to remember the strategies (100%, n=13).  **Factors related to acceptability:**  **Barriers to acceptability**, poor health status | **Weak** |
| UK  Leung [62], 2017 | qualitative | 23 dyads | **cognitive stimulation therapy intervention** (three30-minute sessions weekly for 25weeks)  individual cognitive stimulation therapy intervention program, | Not reported | Home | **Acceptability of psychosocial interventions:**  **Completion rate**  61% (n=14) caregivers completing more than 38 sessions.  **Factors related to acceptability:**  **Barriers to acceptability**  Time constraints | **Moderate** |
| USA  Chee [46], 2007 | RCT | 255 caregivers enrolled;  Intervention group (n=129);  Control group (n=126); | **Intervention group: Skill-Building Intervention (**6 months, six 90-minute home visits/ three 20-minute telephone sessions**)**  Education, problem solving, communication, environmental and task simplification techniques, home modification.  **Control group:** usual care | Therapist | Home | **Acceptability of psychosocial interventions:**  **1)Recruitment and Participation rate**  290 were eligible for study participation, of whom 255 (88%) were willing to participate;  **2) Completion rate**  Of the 255 persons initially enrolled, 188 participated in the 6-month follow-up interview, representing a 26% attrition rate. | **Strong** |
| USA  Callan [70], 2015 | RCT | 60 caregivers;  intervention group (n=27);  control group (n=33) | **Intervention group:**  **cognitive control intervention (**2 months/Internet-based)  provide information about dementia, social support, coping skills training, affective self-management, and healthy sleep practices.  **Control group:** nutrition skills and meal planning. | Nurse | Home | **Acceptability of psychosocial interventions:**  **Completion rate**  74% of participants used APSVAT 18 or more times during the 4-week exposure | **Weak** |
| USA  Zauszniewsk [26], 2016 | Mixed method | 63 caregivers | **Resourcefulness training (RT) intervention** (4 weeks/8 sessions)  included three social (help-seeking) and five personal (self-help) resourcefulness skills training | Graduate students | Community health  centers, churches, and places of business | **Factors related to acceptability:**  **Barriers to acceptability**, the intervention was too time-consuming | **Moderate** |
| USA  Gaugler [71], 2015 | pre-test/post-test | 41 caregivers | **Cognitive**  **Psycho-education program**  (included three 1-hour training modules)  Include dementia education, stress management, etc. | Clinical and scientific experts | Internet-based | **Acceptability of psychosocial interventions:**  **Satisfaction scale**  More than 90% of family caregivers strongly agreed or agreed that CARES for Families was clear, easy to understand, and improved confidence in dementia care;  2)open-ended questions to assess acceptability | **Weak** |
| UK  Woods [64], 2016 | RCT | 488 dyads  intervention group (n=268);  control group (n=219) | **Intervention group:**  **RYCT program intervention** (10 months/10 weekly sessions, 7 monthly sessions)  Include a range of activities including art, cooking, physical re-enactment of memories, singing and oral reminiscence  **Control group:** usual care | Trained facilitators | Community settings | **Acceptability of psychosocial interventions:**  **1) Recruitment and participation rate**  2908 potential participants were considered for inclusion, eligible and available 36% agreed to take part, 488 participated.  **2)Completion rate**  350 dyads completed the study, only 57% of participants attended at least half of the intervention sessions.  **Factors related to acceptability:**  **Barriers to acceptability**, physical complaints, lack of need | **Moderate** |
| Finland  Laakkonen [44], 2013 | RCT | 136 dyads  intervention group (n=67);  control group (n=69) | **Intervention group:**  **Self-management group intervention** (2 months/8 weekly 4-hour sessions)  self- management skills, include self-efficacy, problem-solving skills and peer support.  **Control group:** usual care | Trained professionals, | Day care center | **Acceptability of psychosocial interventions:**  **1) Completion rate**  No one dropped out, participation in these sessions was 93%.  **2) Satisfaction survey**  They were very satisfied with the intervention. 97% caregivers felt that the group rehabilitation was useful, and of the patientswith dementia 53/60 (88%) felt similarly. | **Strong** |
| Finland  Pitkala [45], 2011 | RCT | 210 dyads  Home-based group(n=70);  Group-based group(n=70);  Control group(n=70) | **Intervention group:**  **Exercise intervention (**12 months/ 1-4 hour session twice a week**)**   1. tailored **home-based exercise** twice weekly (n=70); 2) **group-based exercise** in rehabilitation centers twice weekly (n= 70);   **Control group:** with usual care and information of exercise and nutrition (n = 70) | expert of dementia  physiotherapy | Home & rehabilitation centers | **Acceptability of psychosocial interventions:**  **1) Recruitment and participation rate**  94% caregivers had made the decision to participate in the trial  **2) Completion rate**  16 participants (8%) had dropped out | **Strong** |
| Denmark  Søgaard [51], 2014 | RCT | 330 dyads  **Intervention group** (n=163)  **control group** (n=167) | **Intervention group:**  **multicomponent intervention**  7 weeks / 2-hour counseling sessions & 1 education course per week. Include coping strategies, disease education, communication skills, et al.  **Control group:** usual care | Nurse | Home/hospital-based memory clinic | **Acceptability of psychosocial interventions:**  **Completion rate**  6 dyads dropped out in baseline  During the follow up, 66 dyads dropped out. | **Strong** |
| UK  Livingston [47], 2014 | RCT | 260 caregivers  intervention group (n=173);  control group (n=87) | **psychological intervention**( 2 months/8 sessions)  **Intervention group:**  START manual-based, individual coping intervention  **Control group:**  Include medical, psychological, and social support, and is supposed to consist of assessment, diagnosis and information-giving, risk assessment and management. | Therapist | Home | **Acceptability of psychosocial interventions:**  **1) Recruitment and participation rate**  472 carers were screened, 181 (38%) refused to take part, 22 (5%) did not meet inclusion criteria, and 9 (2%) were not contactable, 260 (55%) participated  **2)Completion rate**  Eight (5%) withdrew before any therapy sessions.  196 (75%) finished 24 month follow-up. | **Strong** |
| Germany  Wilz [48],  2016 | RCT | 229 caregivers  Intervention group(n=102)  PMR group：  (n=45)  Control group:  (n=44) | **Intervention group:**  Cognitive Behavioral Therapy intervention (3 months/7 one-hour sessions)  **PMR group**：  Caregivers received training in progressive muscle relaxation; written material with a CD training program for PMR were given to the participants  **Control group:**  untreated | Therapist / psychologist | Home/ telephone | **Acceptability of psychosocial interventions:**  **1)Recruitment and participation rate**  343 caregivers were screened,  259 met the inclusion criteria. Thirty people withdrew from the study prior to baseline assessment;  229 (88.4%) participated  **2) Complete rate**  A total of 182 participants fully completed baseline and 6-month follow-up assessments, whereas 47 (20.5%) caregivers dropped out.  **3) Satisfaction survey**  Caregivers from the CBT group evaluated the telephone setting as very good (71.9%) and 27% as good. | **Strong** |
| USA  Burgio [49],  2003 | two-group comparison design | 118 dyads  intervention group (n=57);  control group (n=61) | **Intervention group:**  **psychosocial intervention** (12 months/one 3-hour sessions, 15one-hour sessions)  skills training program  **Control group:**  minimal support control program | Therapist | Home | **Acceptability of psychosocial interventions:**  **1)Recruitment and participation rate**  289 dyads were screened, 203 were deemed eligible, and 140 were consented to participate.  **2) Completion rate**  There were 118 dyads that completed a 6-month assessment, | **Strong** |
| France  Rotrou [50],  2011 | RCT | 167 dyads  intervention group (n=81);  control group (n=86) | **Intervention group:**  **Cognitive** psycho-educational programme (3 months/an 1-hour session per week)  Include education, problem-solving techniques, emotion-centred coping strategies, behavior management, communication skills, crisis management, practical advice  **Control group:**  waiting list control | Psychologist, geriatrician, psychiatrist, et al. | Not reported | **Acceptability of psychosocial interventions:**  **1)Recruitment and participation rate**  167 dyads screened, 2 dyads did not fulfil inclusion criteria and 8 withdrew their consent.  **2) Completion rate**  141 dyads completed the intervention  115 dyads completed the follow-up | **Strong** |
| Canada  Mohide [65],  1990 | RCT | 60 caregivers  intervention group (n=30);  control group (n=30) | **Intervention group:**  The experimental set of supportive interventions was directed at helping the caregivers enhance caregiving competence and achieve a sense of control in their roles as caregivers  **Control group:**  Usual care | Nurse | Home | **Acceptability of psychosocial interventions:**  **Completion rate**  42 (70%) caregivers completed the trial | **Moderate** |
| Europe  Barbabella [35], 2018 | Mix methods | 118 caregivers | **Web-based psychosocial intervention (3 months/** 1-hour session weekly**)**  Psychological and social support  Coping and reconciliation strategies, education, contact service. | Psychologist, social workers | Internet-based | **Acceptability of psychosocial interventions:**  **1) Recruitment and participation rate**  123 screened, 118 caregivers was enrolled, five participants dropped out due to personal reasons (eg, death of the older person, changed  life circumstances, lack of time)  **2)Completion rate**  94 (79.7%) caregivers used the services offered by the program at least once  **3) Satisfaction survey**  The great majority of participants reported that the Web program was useful for addressing both current and possible future caregiving needs.  **Factors related to acceptability:**  **barriers to acceptability**  Caregivers reported that the main barriers to use of the platform were time constraints, while technical problems and the complexity of the platform were reported as limiting factors | **Moderate** |
| USA  Belle [52] ,  2006 | RCT | 670 caregivers  **Hispanic** **intervention group** (n=106)  **control group** (n=106)  **White** **intervention group**　(n=113)  **Control group** (n=106)  **Black** **intervention group** (n=104)  **Control group** (n=107) | **Intervention group:**  **multicomponent intervention** (6 months/ 12 sessions, included 9 in-home [1.5 hours each] sessions and 3 telephone [half hour each] sessions) /5 structured telephone support group sessions.  included provision of information, didactic instruction, role playing, problem solving, skills training, stress management techniques, and telephone support groups.  **Control group:** mailed caregivers a packet of educational materials and provided 2 brief (15 minute) telephone “check-in” calls | certified interventionists | Home/telephone | **Acceptability of psychosocial interventions:**  **1)Recruitment and participation rate**  995 screened, 670 participated, 642 were enrolled  **2)completion rate**  In the intervention group, 60% completed all 12 sessions;  90% of the 319 caregivers received both telephone contacts in the control group. | **Strong** |
| Hong Kong, China  Kwok [53] ,  2012 | RCT | 102 dyads  **Intervention group** (n=59 )  Control group (n=43) | **Intervention group:**  **Case management (3 months)**  performed functional assessments, advised on coping strategies, skills training and behavioural management | occupational therapist | Home/telephone | **Acceptability of psychosocial interventions:**  **Completion rate**  Of 102 subjects recruited, 10 subjects dropped out. | **Strong** |
| USA  Vickrey [66] ,  2006 | RCT | 408 dyads  **intervention group** (n=238 )  **control group** (n=170) | **Intervention group: care management intervention**  teach problem-solving skills; initiate care plan actions; and send an assessment summary, a problem list, provide supports based on their need.  **Control group:** usual care | care managers | primary care clinics/ internet-based | **Acceptability of psychosocial interventions:**  **1)Recruitment and participation rate**  Of 1043 patients initially identified from claims data and contacted about enrollment through their provider, 91 were ineligible, 308 declined to participate, and 236 did not respond, 408 dyads were enrolled.  **2)completion rate**  Survey response rates were 88% at 12 months and 82% at 18 months | **Moderate** |
| Finland  Eloniemi-Sulkava [54] ,  2009 | RCT | **125 dyads**  **intervention group** (n=63) **control group** (n=62) | **Intervention group: multicomponent support program** (8 sessions/ 2 hours per week)  goal-oriented peer support group meetings, dementia education,  **control group:** usual community care | geriatrician | Home | **Acceptability of psychosocial interventions:**  **1)Recruitment and participation rate**  197 screened, 51 not fulfilling inclusion criteria, 21 refused, 125 participated.  **2)completion rate**  100% completed | **Strong** |
| USA  Mittelman [55] ,  2007 | RCT | **406 caregivers**  **intervention group** (n=203) **control group** (n=203) | **Intervention group: Individual and family counseling (6 sessions/ per week),** The content of the counseling sessions depended on the needs of each spouse caregiver and family.  **control group:** usual care | counselor | Telephone | **Acceptability of psychosocial interventions:**  **1)Recruitment and participation rate**  458 screened, 48 did not meet inclusion criteria, 4 refused , 408 participated  **2)completion rate**  396 (97.5%) completed | **Strong** |
